# Prevalence of Unfilled MB2 Canals and Their Association with Apical Periodontitis: A CBCT-Based Cross-Sectional Study in a German Population

**DOI:** 10.3390/diagnostics16050796

**Published:** 2026-03-07

**Authors:** Maythem Al Fartousi, Christian Ralf Gernhardt

**Affiliations:** 1 Private Practice, Landauer Str. 16, 76185 Karlsruhe, Germany; info@endo-karlsruhe.de; 2 University Outpatient Clinic for Conservative Dentistry and Periodontology, Department of Dentistry, Medical Faculty, Martin-Luther-University Halle-Wittenberg, Magdeburger Strasse 16, 06112 Halle, Germany

**Keywords:** endodontics, molar, maxilla, second mesio-buccal canal, root canal therapy, periapical periodontitis, cross-sectional studies, dental pulp cavity, spiral cone-beam computed tomography, treatment outcome

## Abstract

**Background/Objectives**: The presence of untreated second mesio-buccal canals (MB2) in maxillary first molars is usually associated with endodontic treatment failure. Previous CBCT-based investigations have evaluated the quality of root canal fillings and the prevalence of apical lesions in endodontically treated teeth. However, evidence specifically addressing untreated MB2 canals and their association with apical periodontitis remains limited. Therefore, the aim of this cross-sectional study was to evaluate the prevalence of unfilled MB2 canals in endodontically treated maxillary first molars and their association with apical periodontitis. **Methods**: CBCT scans of 75 patients from an endodontic practice were retrospectively analyzed. Maxillary first molars (teeth 16 and 26) were evaluated for the presence and filling status of root canals (MB1, MB2, palatal, distal) and the presence of periapical radiolucency using the CBCT periapical index. Two calibrated examiners independently assessed all images. The association between unfilled MB2 canals and apical periodontitis was analyzed using chi-square tests, and odds ratios with 95% confidence intervals were calculated. **Results**: The mean patient age was 53.4 ± 15.5 years (range: 14–80). An MB2 canal was present in 84% (63/75) of eligible teeth. Among teeth with an MB2 canal, only 20.6% (13/63) were endodontically filled, while 79.4% remained untreated. Apical periodontitis was observed in 65.3% (49/75) of all teeth. A significant association was found between unfilled MB2 canals and apical periodontitis (*p* < 0.001), with an odds ratio of 0.095 (95% CI: 0.022–0.402), indicating that filled MB2 canals significantly reduced the possible risk of periapical pathology. **Conclusions**: A high prevalence of unfilled MB2 canals was observed in this German population (79.4%). Furthermore, unfilled MB2 canals were strongly associated with apical periodontitis. Therefore, clinicians should utilize all available diagnostic tools, including CBCT and dental microscopes, to maximize MB2 canal identification and improve endodontic treatment outcomes.

## 1. Introduction

The main objectives of root canal treatment are the adequate biomechanical shaping, cleaning and filling of the entire root canal system in a three-dimensional manner. Effective disinfection protocols, including the appropriate use of irrigants such as sodium hypochlorite, ethylenediaminetetraacetic acid, citric acid and chlorhexidine, are essential for successful treatment outcomes, although the potential toxicity of concentrated irrigants must be considered [1,2]. Failure to achieve this inevitably leads to an unfavorable outcome [3]. Several investigations have shown that many biological and treatment-related factors are responsible for endodontic failure, including, but not limited to, persistent bacterial infection [4], inadequate root filling [5] and untreated/omitted canals [6]. Only a few studies have reported failures in root canal treatment due to unfilled canals. These ranged from 12% to 42% in different populations [7,8,9,10]. A recent systematic review and meta-analysis confirmed that missed canals detected via CBCT are significantly associated with post-treatment apical periodontitis (PAP) in root-filled teeth, with the maxillary first and second molars being the most affected teeth due to the high frequency of untreated MB2 canals [11]. Previous CBCT-based investigations conducted in German populations have evaluated the overall quality of root canal fillings and the prevalence of apical radiolucencies following endodontic treatment [12]. However, these studies did not specifically assess untreated MB2 canals or their dedicated association with apical periodontitis in maxillary first molars. Therefore, evidence focusing specifically on untreated MB2 canals and their clinical impact remains limited.

Regarding former investigations, the knowledge, recognition and localization of all existing canals within the root canal system to achieve an optimal prognosis has been widely discussed, and the possible negative effects of untreated canals on the overall treatment outcome have been extensively debated, with overwhelming evidence of unfilled canals in failed cases requiring endodontic intervention [3,4,5,13].

Recent meta-analyses have reported the worldwide prevalence of MB2 canals in maxillary first molars to be approximately 70%, with significant variation among populations ranging from 48% to 97.6% [14]. In older studies [15,16,17,18], conventional intraoral radiography was used to assess apical periodontitis after treatment. Due to the limitations associated with this two-dimensional (2D) method for assessing a three-dimensional object, the localization of untreated canals has been a clinical challenge to date. Cone-beam computer tomography (CBCT), on the other hand, enables precise visualization of a specific tooth in 3D [10]. The prevalence of missing canals in endodontically treated teeth and their relation to apical lesions have been investigated in recent studies in various populations using CBCT [7,8,10], which provided more accurate and reliable results than the conventional two-dimensional method.

Contrary to previous studies evaluating overall endodontic treatment quality, the present study applies a canal-specific and tooth-specific CBCT approach to investigate unfilled MB2 canals in maxillary first molars within a German population, thereby providing clinically focused results on a key factor potentially influencing post-treatment apical periodontitis. Nevertheless, multiple investigations have demonstrated a higher prevalence of apical periodontitis in teeth that have already received root canal treatment [19,20,21,22]. In such situations, the clinical outcome can be adversely affected by deficiencies in the root canal obturation, including inadequate compaction, insufficient apical length, or overextension of filling materials into the periapical region [5,23,24,25]. Evidence from additional studies indicates that a well-sealed coronal restoration plays a critical role in limiting coronal leakage and subsequent reinfection [26,27,28,29]. Post-treatment endodontic pathology is therefore considered multifactorial in origin [30]. A systematic review conducted by Ng et al. (2008) identified four determinants consistently associated with favorable treatment outcomes: the absence of preoperative periapical radiolucency, an obturation terminating within 2 mm of the radiographic apex, a homogeneous root canal filling free of voids, and the presence of an adequate coronal restoration [31]. A recent systematic review on CBCT-assessed treatment outcomes confirmed that treatment success rates appear significantly lower when evaluated by CBCT compared to periapical radiographs, highlighting the higher sensitivity of three-dimensional imaging for detecting persistent periapical pathology [32].

An initial literature review included four recent studies [7,33,34,35]. The studies indicated that the prevalence of untreated MB2 canals is high despite advances in modern endodontics. This is particularly relevant as all studies indicate that unfilled MB2 canals are significantly associated with the occurrence of periapical lesions. The literature search revealed that only a few studies have been conducted in Germany using CBCT to investigate the prevalence of unfilled canals and their association with apical periodontitis. Therefore, the aim of the present clinical and radiographical study was to evaluate the prevalence of missing canals in endodontically treated teeth and their association with apical periodontitis or lesions in a German population using CBCT. Clinically relevant data from the everyday clinical practice of a dental practice specializing in endodontics was used for this purpose. The hypothesis to be tested was that missing treatments of the MB2 is associated with apical periodontitis. Accordingly, the null hypothesis stated that the filling status of the MB2 canal has no statistically significant association with the presence of apical periodontitis.

## 2. Materials and Methods

### 2.1. Study Design

Ultimately, CBCT examinations from 75 patients were retrieved from the patient information system of an endodontic practice. Each scan was evaluated by two independent calibrated and trained examiners (M.A.F and C.R.G.) following a standardized, stepwise screening protocol (see Section 2.3). Both examiners evaluated all images separately and remained blinded to each other’s classifications throughout the process.

The protocol consisted of an initial tooth/root selection (tooth 16 or 26), followed by mandatory root alignment in the coronal, sagittal, and axial planes to achieve a centered view across all three dimensions. Subsequently, tooth/root classification was performed according to predefined parameters, consistently assessed in the same sequence (see below for parameters).

This study was designed as a retrospective investigation based on a random sample. Patients were not pre-selected; instead, inclusion was based solely on existing CBCT data. Ethical approval for the retrospective analysis of anonymized data was obtained from the Ethics Committee of the Faculty of Medicine, Martin-Luther-University Halle-Wittenberg. The committee issued a Clearance Statement for Retrospective, Anonymized Data Analysis under reference number 2025-215. The study was conducted in accordance with the World Medical Association’s Declaration of Helsinki and relevant local regulations. All CBCT scans originated from the existing image archive and had been acquired for various diagnostic purposes unrelated to this study.

All patients were scanned using the same CBCT device: the Carestream Dental–CS 8100 3D Imaging System (Carestream Dental LLC, Atlanta, GA, USA). Inclusion criteria required full-arch scans (field of view “full arch”) with voxel sizes of ≤150 µm, as well as small-volume scans with a 4 × 4 cm field of view. Table 1 summarizes the device specifications and acquisition settings.

Dicom Cleaner™ (version 2016, PixelMed Publishing, Bangor, PA, USA), a software tool that removes all undesired header information from each DICOM slice, was also applied. The anonymized files were subsequently renumbered. In the final step, the files were randomized and renumbered again using the Bulk Rename Utility (version 4.0, TGRMN Software Ltd., London, UK) Special visualization software CS Image SW (version 8.0, Carestream Dental LLC, Atlanta, GA, USA) was used to enable a standardized evaluation method. All samples were analyzed in the coronal, sagittal and axial planes and, where necessary, a noise reduction filter implemented in the visualization software of Carestream Dental (Atlanta, GA, USA) was applied. Regarding the presence of an additional canal in the mesial root, exclusively first molars were considered. Second and third molars, non-restorable root fragments, impacted teeth, deciduous teeth or permanent teeth with immature apices were excluded. Furthermore, scans with partially filled canals or overfilled canals were excluded from the study. Teeth that could not be measured due to image artifacts and image distortions were also excluded. In addition, patients with severe metal artifacts originating from large restorations or implants, which made it impossible to analyze the majority of the existing teeth/roots, were excluded from the study.

All CBCT scans available in practice between 2015 and 2023 were analyzed regarding the inclusion and exclusion criteria. Therefore, a total of 503 CBCT scans were initially screened. After application of the exclusion criteria, 428 scans were excluded, resulting in the final study sample of 75 CBCT datasets from 75 patients included in the analysis. Data analysis was performed between 15 November 2025 and 15 December 2025 and followed the “Strengthening the reporting of observational studies in epidemiology” guidelines (STROBE statement).

### 2.2. CBCT Evaluation: The Used Parameters

Although the primary variable of interest was the MB2 canal, the remaining canals were also evaluated to provide a comprehensive assessment of treatment completeness within multirooted teeth and to allow for contextual interpretation of MB2-related findings. The following parameters were evaluated for patients, first molars and roots: age, gender, tooth (16/26), MB2 (present = 1, not present = 0), MB2_filled (filled = 1, not filled = 0), MB1_filled (filled = 1, not filled = 0), palatal_filled (filled = 1, not filled = 0), distal_filled (filled = 1, not filled = 0), and apical periodontitis (yes = 1, no = 0). Periapical lesions were determined according to the CBCT periapical index score proposed by Estrela et al. (2008) [36]. The absence of periapical pathology was coded as 0 and corresponded to score 0, indicating preserved and intact periapical osseous structures. Conversely, the presence of pathology was coded as 1 and included scores ranging from 1 to 5, which were characterized by a periapical radiolucency exceeding 0.5 mm in diameter. Due to the cross-sectional nature of the study, differentiation between healing and active lesions was not feasible. Spatial alignment of the axial, coronal, and sagittal planes was achieved using a uniform image screening protocol applied consistently across all datasets, enabling verification of root canal length measurements in both the coronal and sagittal dimensions. Because minor discrepancies between these views can arise from anatomical variations in apical root canal morphology, the most conservative (i.e., least favorable) measurement was ultimately selected following comprehensive assessment of all imaging planes. Table 2 shows the extracted variables including the different codes.

### 2.3. Statistical Analysis

All collected data were imported into the data management software Excel (version Microsoft Excel 365, Microsoft Corporation, Redmond, WA, USA) and the SPSS software (version 27; IBM SPSS Statistics, Chicago, IL, USA). Regarding the study design, this study based on retrospectively available CBCT datasets. Therefore, an a priori sample size calculation was not possible. However, a post hoc power analysis was conducted based on the observed effect size of the association between MB2 filling status and apical periodontitis. The calculated statistical power exceeded 80%, indicating that the used sample size of 75 CBCT scans was sufficient to detect the observed association.

#### 2.3.1. Initial Assessment of Intra-Observer Reliability

Prior to the first evaluation of CBCT cases, intra-observer reliability was assessed. A randomly selected subset of 20 CBCT scans was re-evaluated four weeks after the first evaluation under identical viewing conditions, with the examiner blinded to the initial assessments. Agreement between evaluations was calculated using Cohen’s kappa statistics.

#### 2.3.2. Statistical Analysis of the Evaluated Data

Analysis was performed after structured and anonymized extraction from the practice’s patient information system. The primary outcome was the prevalence of periapical lesions, while the predictive variable was adequate root filling of the MB2 of the first molar. The odds ratio and the lower and upper limits of the 95% confidence intervals (CIs) were calculated using the data. The odds ratio (OR) was calculated to analyze the probability of apical lesions occurring in teeth with unfilled canals compared to teeth with treated canals. Paired analyses were performed to analyze the differences in the prevalence of apical periodontitis depending on adequate filling (MB1, MB2, distal, palatal). A *p*-value of < 0.05 was considered significant for all groups compared.

## 3. Results

### 3.1. Intra-Observer Reliability

Regarding both calibrated examiners, the evaluation showed an excellent intra-observer agreement (Cohen’s κ ≈ 0.90). Inter-observer agreement across all parameters reached 100%, eliminating the need for consensus discussion.

### 3.2. Descriptive Statistics

**Figure 1:**
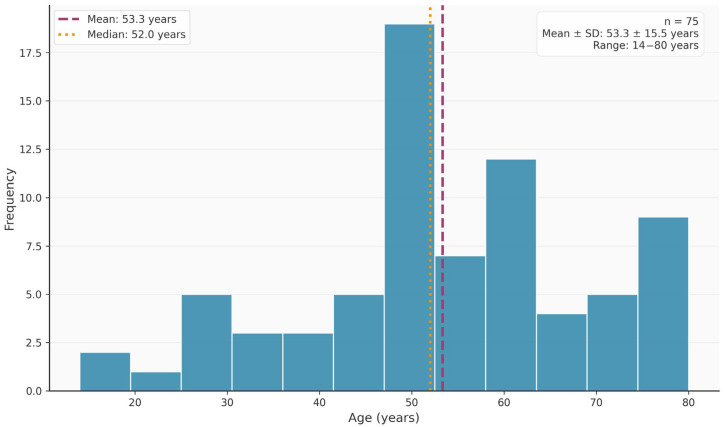
Age distribution in the cohort.

A total of *n* = 75 patients were included. Of these, *n* = 36 (48.0%) were female and *n* = 39 (52.0%) were male. With regard to the first molars, *n* = 36 (48.0%) were 16s and *n* = 39 (52.0%) were 26s. The mean age in the cohort was 53.35 ± 15.48 years and ranged from 14 to 80 years (Figure 1).

Table 3 shows the distribution of tooth-related variables. An MB2 canal was present in 63/75 (84%) of the teeth. Teeth 16 and 26 did not differ significantly in terms of the presence of the MB2 canal (*p* = 0.320). Thirteen of the 63 MB2 canals (20.6%) were endodontically filled. In contrast, 69/75 MB1 canals were endodontically filled. The palatal canal was most frequently filled endodontically (75/75; 100%), followed by the distal canal (68/75; 90.7%). Apical periodontitis was observed in 49/75 (65.3%) of the teeth.

**Figure 2:**
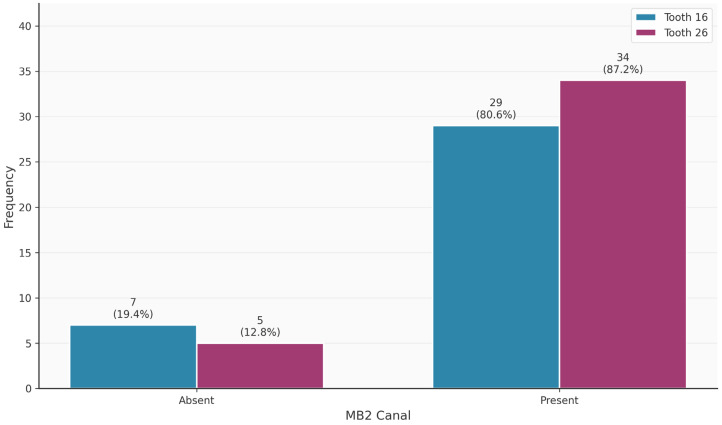
Distribution of the variable “MB2 present” between teeth 16 and 26.

**Figure 3:**
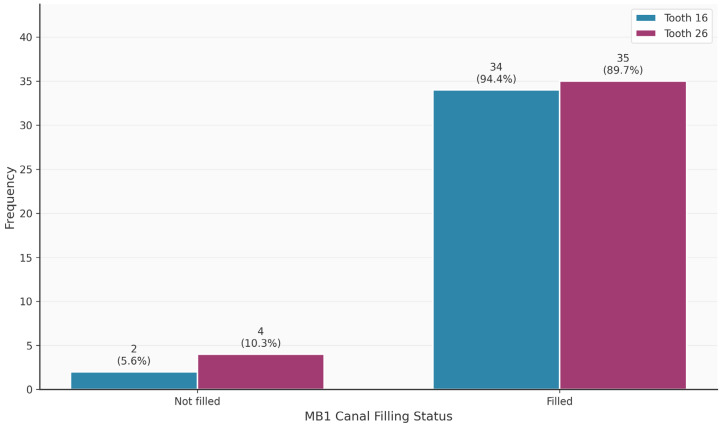
Distribution of the variable “MB1 filled” between teeth 16 and 26.

**Figure 4:**
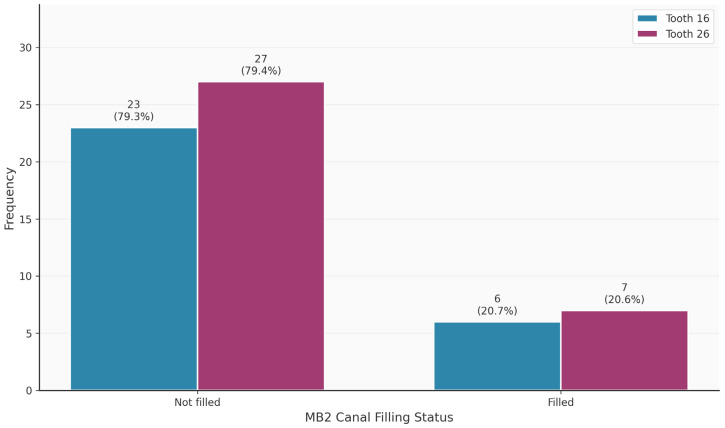
Distribution of the variable “MB2 filled” between teeth 16 and 26.

**Figure 5:**
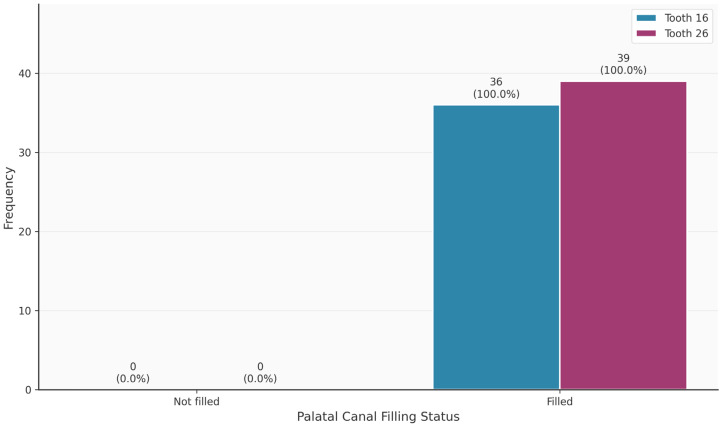
Distribution of the variable “palatinal filled” between teeth 16 and 26.

**Figure 6:**
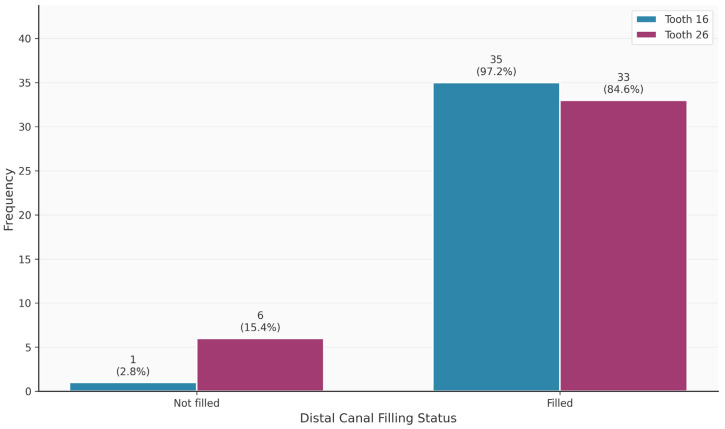
Distribution of the variable “distal filled” between teeth 16 and 26.

**Figure 7:**
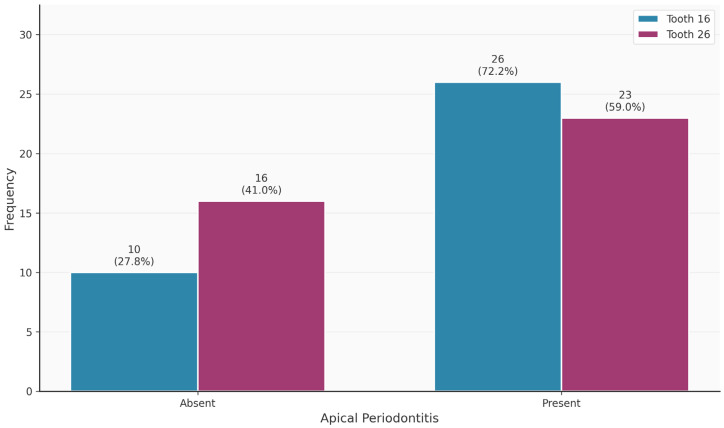
Distribution of the variable “apical periodontitis” between teeth 16 and 26.

Figure 2, Figure 3, Figure 4, Figure 5, Figure 6 and Figure 7 show the distribution of study variables between teeth 16 and 26. None of the study variables for the outcome analysis were significantly different between teeth 16 and 26.

### 3.3. Predictive Analysis

**Figure 8:**
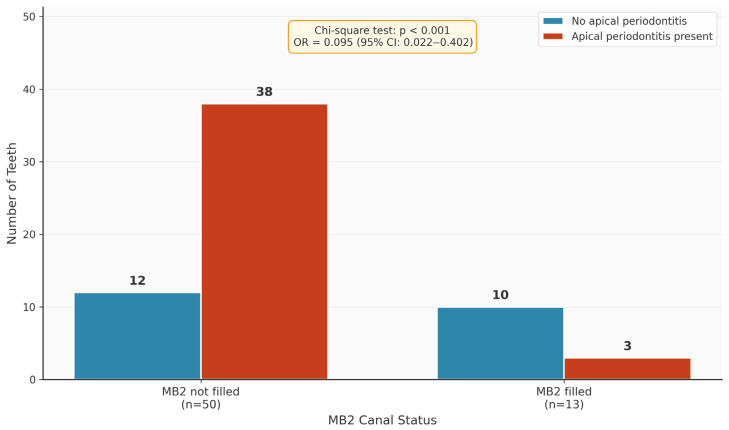
Graphical representation of the distribution of the variable “apical periodontitis” depending on the variable “MB2 filled”.

The next step was to investigate whether the prevalence of unfilled canals was associated with apical periodontitis. Figure 8 and Table 4 show the distribution of the variable “apical periodontitis” as a function of the variable “MB2 filled”. A significant difference in distribution was found (*p* < 0.001). The MB2 canals of teeth with apical periodontitis were thus significantly less likely to be endodontically filled. The odds ratio was 0.095 (95% confidence interval: 0.022–0.402). Filled MB2 canals thus led to a significant reduction in the risk of apical periodontitis. Neither gender (*p* = 0.163), tooth (16 versus 26) (*p* = 0.286) nor age (*p* = 0.401) were significant predictive variables for the occurrence of apical periodontitis.

**Figure 9:**
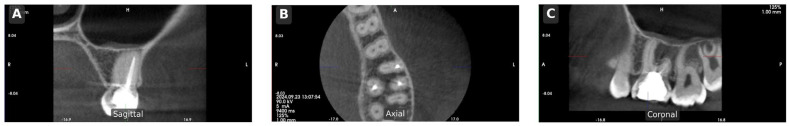
MB2 untreated, no apical periodontitis. Representative cone-beam computed tomography (CBCT) images of a maxillary first molar with an untreated MB2 canal and absence of apical periodontitis. (**A**) Sagittal view showing the root-filled mesio-buccal root. (**B**) Axial view demonstrating the presence of an unfilled MB2 canal adjacent to the filled MB1 canal. (**C**) Coronal view displaying intact periapical bone structures without evidence of periapical radiolucency.

**Figure 10:**
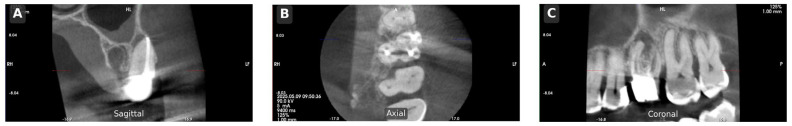
MB2 untreated, with apical periodontitis. Representative cone-beam computed tomography (CBCT) images of a maxillary first molar with an untreated MB2 canal and presence of apical periodontitis. (**A**) Sagittal view demonstrating periapical radiolucency at the mesio-buccal root apex. (**B**) Axial view showing the unfilled MB2 canal alongside the treated MB1 canal. (**C**) Coronal view revealing extensive periapical bone loss associated with the mesio-buccal root.

Representative CBCT images illustrating these findings are shown in Figure 9 and Figure 10. Figure 9 demonstrates a case with an untreated MB2 canal but without signs of apical periodontitis, whereas Figure 10 shows a case where the missed MB2 canal is associated with distinct periapical radiolucency. These examples visually underscore the clinical relevance of identifying and treating the MB2 canal to prevent periapical pathology.

## 4. Discussion

Numerous investigations conducted by independent research groups have consistently demonstrated that inadequately executed root canal obturation is strongly associated with an increased prevalence of periapical periodontitis [19,27,37,38,39,40,41,42,43,44]. Previously published research has demonstrated that cone-beam computed tomography provides superior sensitivity for identifying periradicular alterations when compared with conventional two-dimensional radiographic methods [36,43,44]. A recent retrospective CBCT analysis from Iraq confirmed that the vast majority of teeth with previous root fillings presented with apical periodontitis, with detection risk being significantly higher in root-filled teeth with perforation, non-homogeneous, and underfilled root canal fillings [45].

In an investigation by Estrela and colleagues involving the evaluation of 1425 teeth that had undergone endodontic therapy, three distinct imaging modalities were compared [36]. The authors reported detection rates of periapical pathology of 17.6% when using panoramic imaging, 35.3% with intraoral periapical radiography, and 63.3% when cone-beam computed tomography was employed [36]. The regarded differences were described as statistically significant. The authors concluded that the CBCT technique has a higher sensitivity in identifying periapical lesions and that conventional radiographs tend to underestimate the prevalence of lesions, mainly due to false-negative cases. One factor suggesting this conclusion is the fact that approximately 30-50% bone loss is required for the lesion to be detected on conventional radiographs [36]. However, it is important to note that overdiagnosis of periapical periodontitis in previously root-canal-treated teeth assessed by CBCT has also been reported [46]. A 2-year prospective study comparing CBCT and digital periapical radiography found that CBCT-based evaluation resulted in negative treatment outcomes for 10 more root canals than the radiograph-based result, confirming the higher sensitivity of CBCT in detecting periapical lesions [47].

Furthermore, inter-examiner analyses show that CBCT imaging is a reliable and reproducible method for examining the prevalence of periapical lesions. The overall prevalence of periapical lesions detected in the present study was 65.3%, which is much higher than the range (1.4–15.1%) reported in previous studies using panoramic radiographs [20,42,48] or CBCT images [38,49]. One reason for this is the fact that we only considered root-canal-filled teeth in this study, as these are more likely to be associated with periapical lesions. The root canal system exhibits considerable anatomical variation, which poses challenges not only for canal detection but also for adequate cleaning and obturation [50]. Apical periodontitis was observed in 98% of teeth with untreated or insufficiently treated canals, which was shown to be of significantly higher frequency compared to teeth with all canals completely treated [8]. In a recent CBCT-based study from Chile, 70% of maxillary molars with apical periodontitis had missed MB2 canals, demonstrating a statistically significant association (*p* < 0.0001) [51]. A dentist’s lack of clinical experience regarding the anatomy and complexity of the root canal system, especially in first upper molars, can lead to canals being overlooked during root canal treatment [52]. These canals serve as a pool for microorganisms, which are the main cause of the development or persistence of apical periodontitis [53] and can have a negative impact on the prognosis [54,55]. Several studies have already impressively demonstrated a connection between root-filled teeth and apical periodontitis [21,42,48]. A recent Saudi Arabian CBCT study reported that apical periodontitis was highly associated with missed canals, with an overall prevalence of 90% among teeth with missed canals, reaching 100% in mandibular teeth [56].

The endodontic literature indicates that a high number of unfilled canals require endodontic retreatment. Hoen and Pink found unfilled canals in 42% of all teeth that were treated [9], while Karabucak et al. estimated the overall prevalence of unfilled canals at 23% [10] and Costa et al. and Baruwa et al. found a relatively low prevalence of 12% [7,8]. A recent Iranian CBCT study reported that MB2 had the highest prevalence of untreated canals, and the presence of untreated canals significantly increased the risk of expansion and/or destruction of periapical tissues [57]. In the present study, the overall frequency of unfilled canals in endodontically treated teeth was 79.4% for MB2 canals, 8% for MB1 canals, 0% for palatal canals and 9.3% for distal canals. This is consistent with a recent study that found untreated MB2 canals in 83.5% of examined mesio-buccal roots, attributing this high rate to the use of small voxel sizes (100 µm) in CBCT imaging [58]. The assessment of additional canals was intended to characterize overall treatment status rather than to establish a direct relationship between these canals and MB2 findings. The difference between the existing studies could be due to the sample, methodology and the population. However, the results confirm the same finding to a certain extent: unfilled canals are still common in clinical practice and represent a risk factor for apical lesions.

Furthermore, the absence of root filling material within an identifiable MB2 canal might indicate incomplete instrumentation, disinfection and obturation of the root canal system and may therefore be interpreted as a radiographic marker of insufficient treatment quality rather than definitive evidence of treatment failure. Accordingly, the observed relationship should be interpreted as an association between treatment adequacy and periapical status at the time of evaluation, without implying causality. However, in multirooted teeth, periapical radiolucencies cannot be definitively attributed to a single untreated canal based solely on cross-sectional CBCT assessment. Therefore, MB2 canals were considered the primary analytical variable of interest, and the observed findings describe an association between the presence of untreated MB2 canals and periapical status without implying that the MB2 canal alone represents the source of the lesion. It is important to clarify that this study did not evaluate the length of the root canal obturation. Insufficient cleaning, shaping, and disinfection of the apical portion of the canal, coupled with inadequate apical sealing, can result in increased bacterial colonization and a higher likelihood of developing apical periodontitis. Optimal root canal obturation, ideally terminating within 0–2 mm of the radiographic apex [31], has been associated with a reduced incidence of periapical lesions [25,59]. Another study, which used different root filling intervals, concluded that the prevalence of periapical lesions was lower in roots filled within 1-2 mm of the apex, followed by 0 mm from the apex and more than 2 mm shorter than the apex [60]. The same study reported that the prevalence of periapical lesions is higher in molars. We did not find any difference in the prevalence of apical periodontitis between both sides of the upper arch, between teeth 16 and 26.

Furthermore, when comparing well-filled (4.0%) and overfilled (45.4%) canals, a previous study found a similar prevalence of periapical lesions [59]. Another study found that the extrusion of used dental materials (sealer or gutta-percha) extended healing of periapical tissues [60]. It has been shown that overfilling can cause irritations and the recruitment of inflammatory cells into the periapical tissue [61], especially when using non-biocompatible or formaldehyde-containing sealers [62,63].

The clinical identification of MB2 canals remains challenging, even with modern technology. A recent study investigating variables affecting MB2 localization found that patient gender, tooth type, and treatment modality play pivotal roles, with the availability of preoperative CBCT imaging being associated with a heightened ability to locate the MB2 canal [64]. Furthermore, the use of dental operating microscopes combined with ultrasonic instrumentation has been shown to increase MB2 detection rates to 86% compared to 79% with direct vision alone [65].

The most important limitation of our cross-sectional study, apart from the sample size, is the type of study, in which the teeth were examined at a specific point in time and thus the information about the course of apical periodontitis is not precisely known, meaning that no cause-and-effect relationship can be established. Regarding the retrospective cross-sectional design of the study, it was not possible to establish a temporal baseline. However, this limitation is inherent to observational cross-sectional CBCT studies, as pre-treatment or immediate post-treatment baseline scans are typically unavailable due to ethical considerations related to radiation exposure. In addition, many factors can influence the treatment outcome, including applied techniques, used materials and instruments, the achieved asepsis, the used disinfection protocol, the obturation protocol used, the preoperative status of the tooth, and the expertise of the clinicians [56]. CBCT imaging is a valuable clinical tool in the assessment of periapical lesions [66], as it provides three-dimensional information and has higher sensitivity than radiographs when evaluating changes in dental hard tissues [67]. A recent 4-year longitudinal study demonstrated that when the radiolucency changed by 20% or more in volume on CBCT scans at 1 year after root canal treatment, reversal of the radiographic healing tendency was rare, supporting the use of CBCT for early clinical decision-making [68]. In this study, each tooth was systematically assessed in axial, sagittal, and coronal planes to optimize measurement reliability. All imaging datasets were obtained for routine clinical care rather than for research purposes, thereby ensuring that patients were not subjected to additional radiation exposure. To enhance the statistical power and representativeness of the sample, both full-arch scans and 4 × 4 field-of-view acquisitions were included in the analysis, allowing for a broader inclusion of clinical cases. However, the use of small FOV scans in combination with full-arch examinations could affect comparability with other studies. Targeted endodontic imaging is primarily utilized for therapeutic evaluation, rendering it more effective in identifying periapical lesions and endodontic complications than full-arch scans. Although this study analyzed a randomly selected sample, the results provide meaningful insight into the prevalence of periapical pathology in routine clinical settings, thereby enhancing external validity. These findings may guide the formulation of optimized strategies for the prevention, management, and monitoring of periapical disease. Importantly, a robust correlation between cone-beam computed tomography observations and histopathological outcomes remains underexplored, underscoring the necessity for further longitudinal studies to validate imaging-based diagnostic accuracy.

## 5. Conclusions

In the present study, the frequency of apical lesions in teeth with unfilled canals was high (84%), with most unfilled canals identified as MB2 canals (79.4% unfilled). All available measures, including CBCT and dental microscopes, should be used to maximize the identification of MB2 canals and thus reduce the prevalence of apical lesions. Longitudinal studies and translational studies with large case numbers are needed to further evaluate predictive factors for apical periodontitis and to investigate the effects of unfilled canals on periapical bone loss.

## Figures and Tables

**Table 1:** Overview of the features of the CBCT device used.

CBCT Model	Brand	Voxel Size (µm)	FOV (Small/Full Arch)
CS 8100 3D	Care Stream	75–150	4 × 4 cm/8 × 8 cm

**Table 2:** List of study variables. The codes and calculation formulas are shown in the “Formula” column. These were used to facilitate statistical analysis.

Variable	Data	Variable Type	Formula
***Patient ID***	In digits	Nominal	-
***Gender***	In digits	Nominal	0 = male 1 = female
***Age***	In digits	Metric	-
***Tooth (16, 26)***	In digits	Nominal	0 = 16 1 = 26
***MB2***	In digits	Nominal	0 = not available 1 = present
***MB2_filled***	In digits	Nominal	0 = not filled 1 = filled
***MB1_filled***	In digits	Nominal	0 = not filled 1 = filled
***Palatal_filled***	In digits	Nominal	0 = not filled 1 = filled
***Distal_filled***	In digits	Nominal	0 = not filled 1 = filled
***Apical periodontitis***	In digits	Nominal	0 = no 1 = yes

**Table 3:** Distribution of study variables for outcome analysis.

		Number	Number in %
MB2 (present = 1, absent = 0)	0	12	16.0
	1	63	84.0
	Total	75	100.0
MB2_filled (filled = 1, not filled = 0)	0	50	79.4
	1	13	20.6
	Total	63	100.0
MB1_filled (filled = 1, not filled = 0)	0	6	8.0
	1	69	92.0
	Total	75	100.0
palatinal_filled (filled = 1, not filled = 0)	1	75	100.0
	Total	75	100.0
distal_filled (filled = 1, not filled = 0)	0	7	9.3%
	1	68	90.7%
	Total	75	100.0
Apical periodontitis (yes = 1, no = 0)	0	26	34.7
	1	49	65.3
	Total	75	100.0

**Table 4:** Nominal distribution of the values "apical periodontitis” depending on the variable “MB2 filled”.

		MB2_Filled (Filled = 1, Not Filled = 0)		Total
		0	1	
Apical periodontitis (yes = 1, no = 0)	0	12	10	22
	1	38	3	41
Total		50	13	63

## Data Availability

The data presented in this study are available on request from the corresponding author. Because the dataset contains anonymized clinical imaging information, access can be granted to qualified researchers in accordance with institutional and ethical guidelines.
